# Inverted ILM-flap techniques variants for macular hole surgery: randomized clinical trial to compare retinal sensitivity and fixation stability

**DOI:** 10.1038/s41598-020-72774-1

**Published:** 2020-09-28

**Authors:** Andrea Cacciamani, Aldo Gelso, Marta Di Nicola, Fabio Scarinci, Guido Ripandelli, Ciro Costagliola, Tommaso Rossi

**Affiliations:** 1 IRCCS - Fondazione Bietti, Via Livenza, 3, 00198 Rome, Italy; 2 Villa Dei Fiori, Acerra, Naples, Italy; 3 Department of Medical, Oral and Biotechnological Sciences, “G. D’Annunzio” University Chieti-Pescara, Chieti, Italy; 4 Department of Medicine and Science of Ageing, School of Hygiene and Preventive Medicine, “G. D’Annunzio” University Chieti-Pescara, Chieti, Italy; 5 Department of Medicine and Health Sciences “Vincenzo Tiberio”, University of Molise, Campobasso, Italy; 6 IRCCS Ospedale Policlinico San Martino, Genoa, Italy

**Keywords:** Diseases, Medical research

## Abstract

To report closure rate, Best Corrected Visual Acuity (BCVA), Retinal Sensitivity (RS) and Fixation Stability (FS) of idiopathic Macular Holes (MH) randomized to Cover Group (CG) or Fill Group (FG) of the Inverted Internal Limiting Membrane (ILM) flap surgical procedure. Twenty-eight patients were randomized (1:1) to receive a vitrectomy with either Cover or Fill ILM flap technique. All patients underwent BCVA, RS and FS assessment at baseline, 1-month and 3-months after surgery. MH closed in all patients. BCVA rose from 20/100 (baseline) to 20/33 (1-month) in both groups, to 20/28 in CG versus 20/33 in FG (3-months) (*p* < 0.05). The central 4° RS rose from 11.5 and 12 dB to 19 and 19.5 dB (1-month) and to 22 and 20 dB (3-months), respectively, in CG and FG (*p* < 0.001). The central 10° RS rose from 11 and 15 dB to 22 and 20 dB (1-month) and to 23 and 20 dB (3-months), respectively, in CG and FG (*p* < 0.001). FS increased significantly more in CG. CG improved significantly more than FG in terms of BCVA, RS and FS. The average MH diameter was relatively small (397 µm); larger MHs may behave differently.

Trial registration: Trial Registry: https://www.clinicaltrials.gov; Identifier: NCT04135638. Registration date 22/10/2019.

## Introduction

Macular Hole (MH) became a mendable condition in 1991 when Kelly and Wendel introduced Pars Plana Vitrectomy (PPV) as a treatment achieving a 50% success rate in their pivotal series^1^. Success rate rose ever since to exceed 90% nowadays, due to instrumentation and surgical technique refinement, including the introduction of Internal Limiting Membrane (ILM) inverted flap and the many modifications proposed^2^.

In a previous study, we compared two variants of the ILM inverted flap technique, named because of the different use of the tissue: “Cover” when the ILM flap is everted over the MH gap in a single layer, “Fill” when the ILM is folded into multiple layers within the MH^3^. The two techniques proved equally effective in closing MHs but “Cover” appeared to confer a faster recovery and slightly better visual outcome, whereas “Fill” allowed the closure of larger MHs. Up to date, fixation and retinal sensitivity were not investigated.

Microperimetry is a diagnostic strategy conceptually similar to visual field testing, capable of measuring retinal differential sensitivity to luminous stimuli of various dimension and luminance, projected onto the retinal surface under direct ophthalmoscopic control. Fixation point can also be located on the retinal surface and its stability evaluated throughout the course of examination^4^. Although many authors used microperimetry to assess retinal sensitivity after MH surgery, none specifically compared variants of ILM inverted flap^5, 6^.

Purpose of the present paper is to report retinal differential sensitivity and fixation stability as well as anatomic and visual outcomes of patients operated on for idiopathic MH and randomly assigned to Fill or Cover Groups.

## Results

Overall 28 patients were included in the study: each group comprised 14 patients that did not differ significantly in terms of age and gender (Table 1). All enrolled patients completed the study. Baseline characteristics also appeared well balanced and there was no significant difference in BCVA, ellipsoid zone interruption line, MH width, fixation stability (Table 1).

**Table 1:** Baseline characteristics of patients.

Variable	Cover group (n = 14)	Fill group (n = 14)	*p *value
**Gender, n (%)**			0.702^a^
Female	7 (50.0)	9 (64.3)	
Male	7 (50.0)	5 (35.7)	
Age (year), *median (IQR)*	67.5 (65.2–72.7)	66.5 (60.2–68.0)	0.269^b^
MH width (µm), *median (IQR)*	351.0 (310.0–438.2)	456.0 (297.8–535.5)	0.246^b^
Ellipsoid line interruption width (µm), *median (IQR)*	739.5 (603.5–932.0)	867.0 (694.0–932.8)	0.427^b^
BCVA (LogMAR), *median (IQR)*	0.2 (0.1–0.2)	0.2 (0.1–0.3)	0.657^b^
**Fixation stability, n (%)**			0.280^a^
0	5 (35.7)	9 (64.3)	
1	3 (21.4)	1 (7.1)	
2	6 (42.9)	4 (28.6)	

Fixation stability: 0 = stable, 1 = relatively (stable, 2 = unstable).

^a^Chi-squared test.

^b^Mann–Whitney U test cover group versus fill group.

Macular hole closed anatomically in 14/14 patients in both groups after surgery. No cases suffered capsular tears or disinsertion.

Median BCVA and 4° and 10° retinal sensitivity of the two groups at baseline, 1 and 3 months is reported in Table 2.

**Table 2:** Median values (interquartile range) of BCVA (logMAR), MAIA 4° and MAIA 10° stratified, and fixation stability frequencies (%) according to the surgical technique with the *p *values derived from ranked based model for differences between the surgery techniques, follow-up time points and interaction between surgery technique and time (surgery × time).

	Cover group			Fill group			*p* value		
	Baseline	1 month	3 months	Baseline	1 month	3 months	Surgery type	Time	Interaction
BCVA (logMAR)	0.70 (0.70–1.07)	0.22 (0.15–0.28)	0.15 (0.09–0.20)	0.70 (0.52–1.08)	0.22 (0.22–0.49)	0.22 (0.11–0.40)	0.246	< 0.001	0.047
MAIA 4° (dB)	11.50 (4.75–13.50)	19.00 (16.50–24.25)	22.00 (20.00–22.75)	12.00 (6.00–15.00)	19.50 (15.75–22.75)	20.00 (17.25–22.50)	0.811	< 0.001	0.044
MAIA 10° (dB)	11.15 (7.30–14.88)	22.78 (19.90–24.88)	23.50 (22.27–25.48)	15.15 (11.07–21.02)	20.15 (17.02–22.93)	20.90 (17.20–24.00)	0.430	< 0.001	< 0.001
**Fixation stability**									
0	5 (35.7)	12 (85.7)	13 (92.9)	9 (64.3)	8 (57.1)	8 (57.1)	0.603	0.003	0.006
1	3 (21.4)	0 (0)	0 (0)	1 (7.1)	5 (35.7)	5 (35.7)			
2	6 (42.9)	2 (14.3)	1 (7.1)	4 (28.6)	1 (7.2)	1 (7.2)			

BCVA, best corrected visual acuity; 0, stable fixation; 1, relatively unstable fixation; 2, unstable fixation.

**Figure 1:**
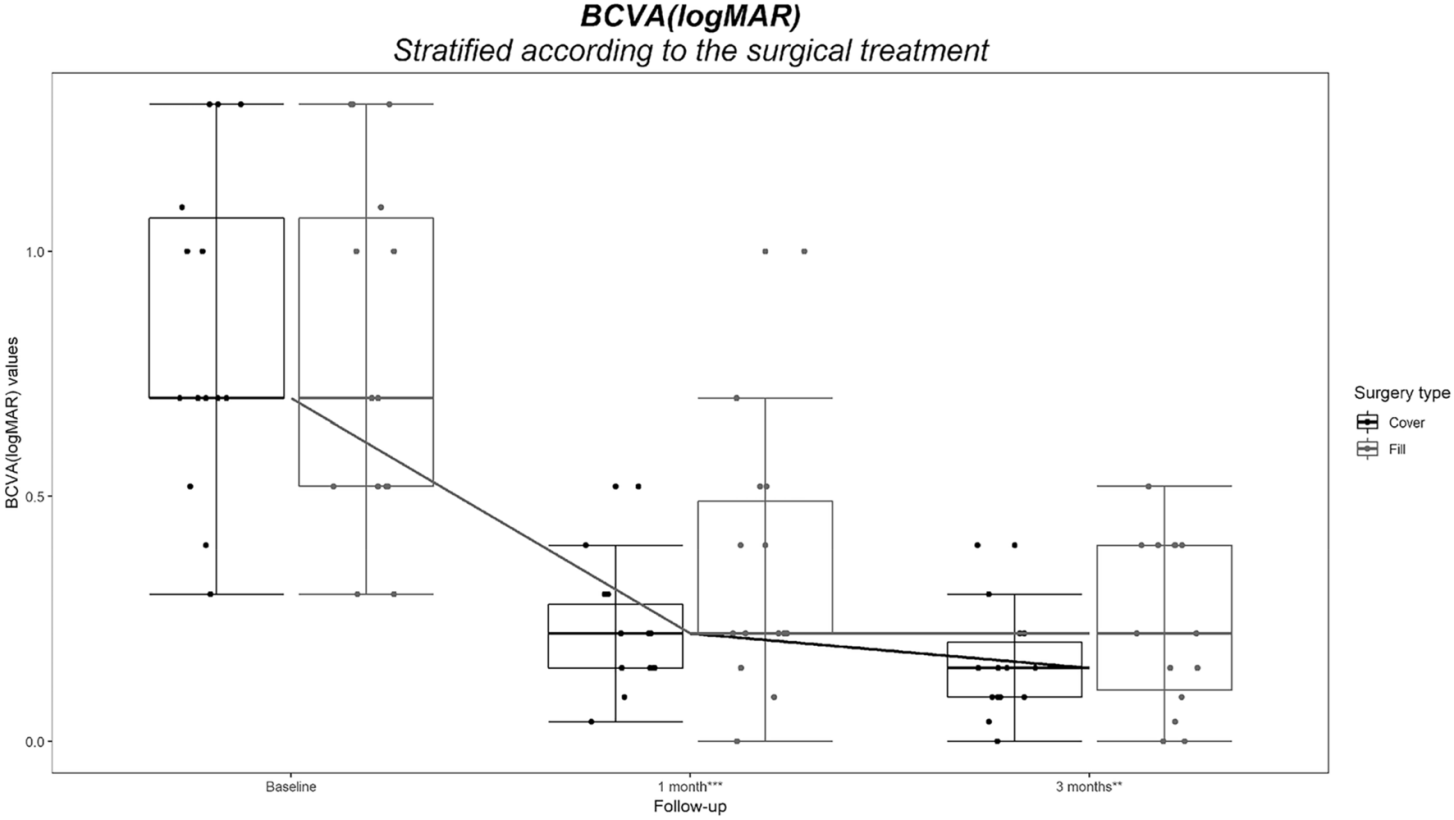
Best corrected visual acuity trend of cover and fill groups at baseline, 1-month and 3-months follow-up. The difference at 3-month follow-up is statistically significant (*p* < 0.001). (GIMP free software, version number GIMP 2.10.18, released 2020-02-24, URL https://www.gimp.org).

Visual acuity and retinal sensitivity within the central 4° and 10° improved significantly in both Cover and Fill groups at month 1 and 3 compared to baseline (Fig. 1 and Table 2; *p* < 0.001 in all cases).

**Figure 2:**
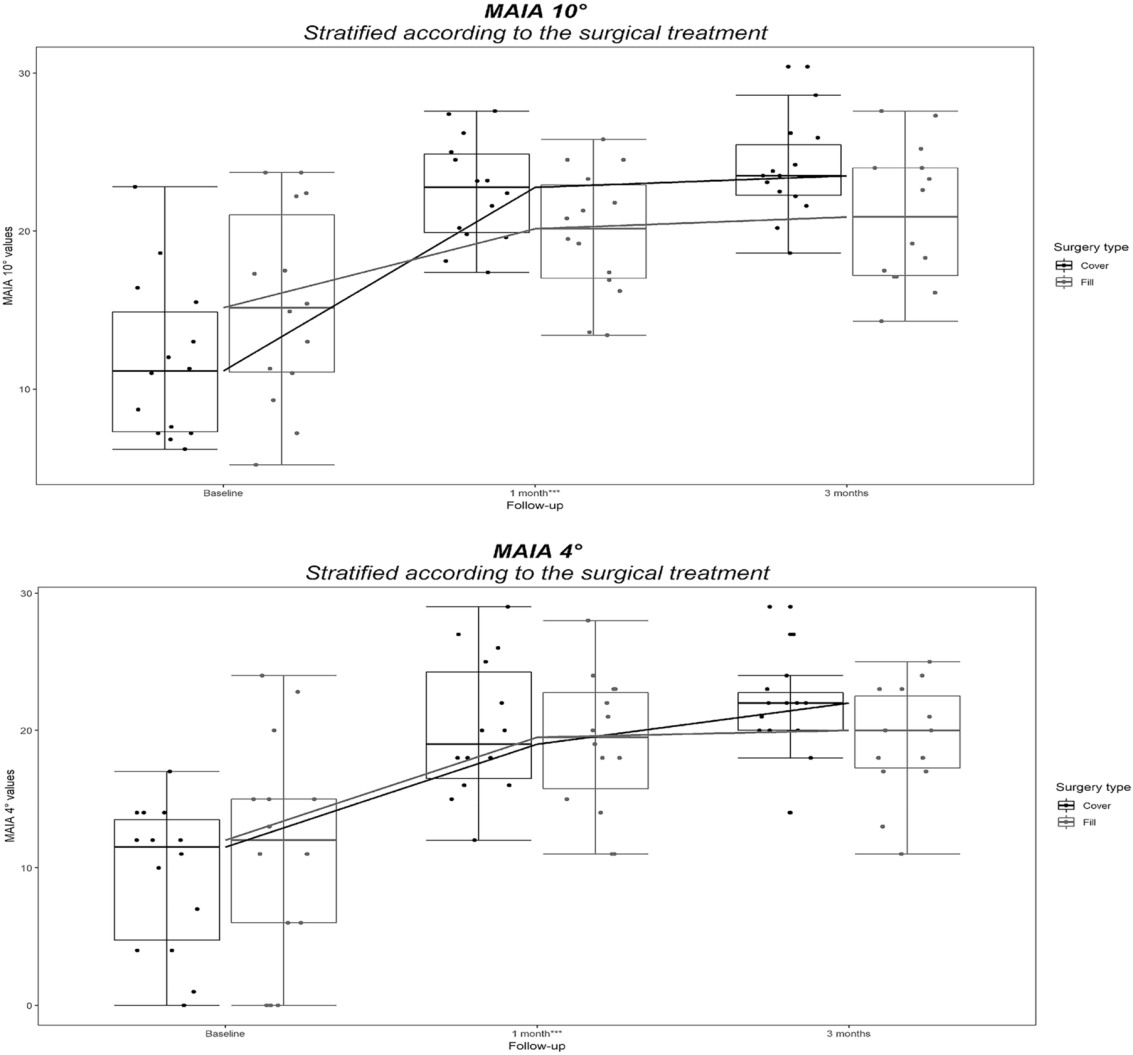
Retinal Sensitivity within the central 4° and 10° trend of Cover and Fill Groups at baseline, 1-month and 3-months follow-up. The difference at 3-month follow-up is statistically significant (*p* < 0.001) (GIMP free software, version number GIMP 2.10.18, released 2020-02-24, URL https://www.gimp.org).

The Cover Group improved more than the Fill Group in terms of BCVA (Table 2, Fig. 1), retinal sensitivity (Table 2, Fig. 2) and fixation stability (Table 2); 8/14 (57.1%) of patients in Cover Group versus 4/14 (28.6%) in Fill Group gained a stable fixation by the end of the third month of follow-up. All patients classified as 1 (relatively stable) or 2 (unstable; see Table 2) at baseline in Cover Group were reclassified as 0 (stable) at 3 months, while none of the patients classified as 1 or 2 in Fill Group reached a stable fixation at 3 months follow-up (Table 2).

**Figure 3:**
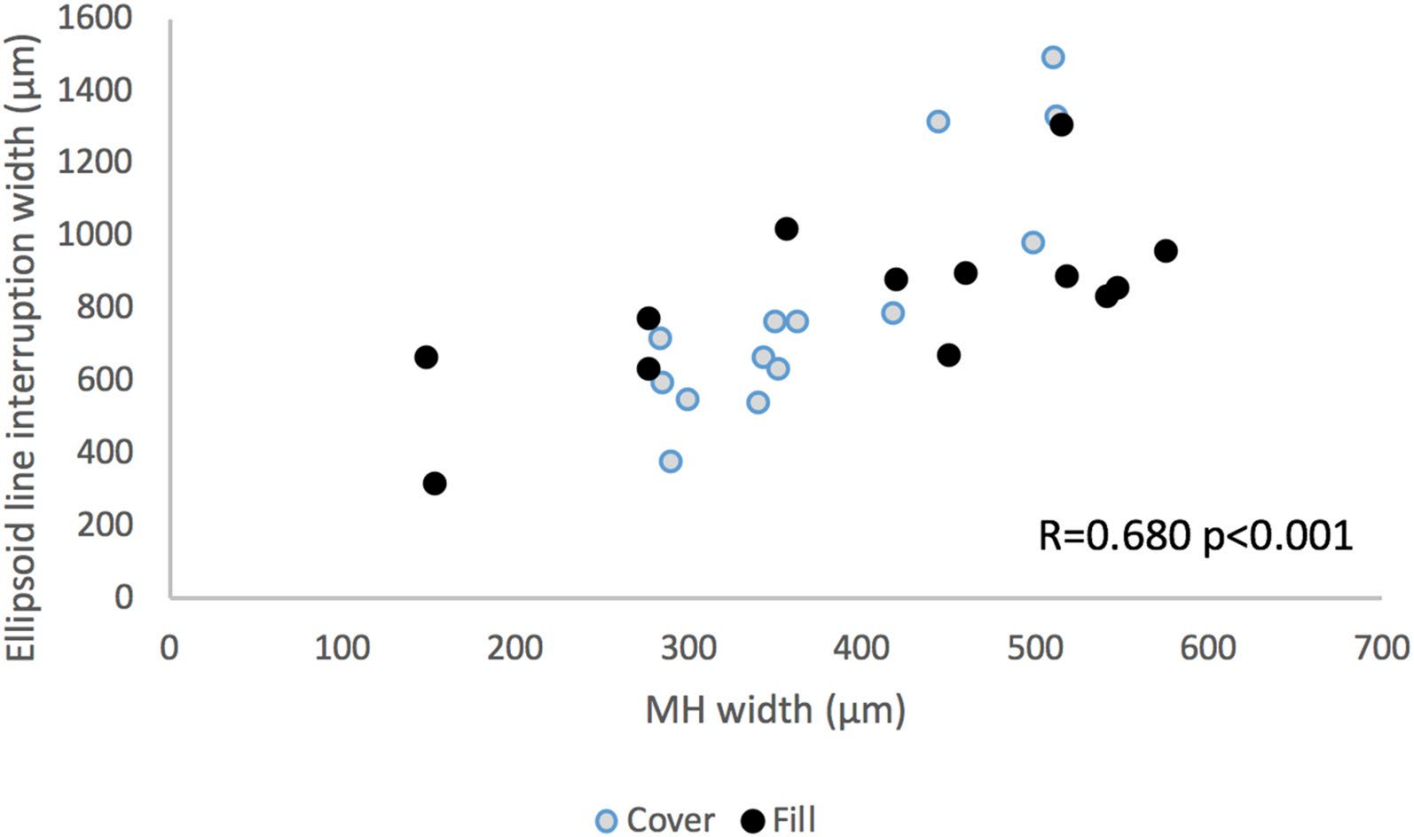
Scattergram showing Macular Hole diameter versus IS/OS line defect width. (GIMP free software, version number GIMP 2.10.18, released 2020-02-24, URL https://www.gimp.org).

MH diameter and ellipsoid zone defect correlated significantly: on average ellipsoid zone defect was 1.5 times larger than the MH maximum diameter (Fig. 3).

Macular hole size of previously reported surgical series is significantly smaller than present series (Supplementary Table S1)^3^. The ICC value for measurements was 0.902.

No important adverse events have occurred.

## Discussion

Macular hole surgery outcomes greatly improved over the past decade thanks to instrumentation and technique refinements, reaching anatomic success rates exceeding 90%. The introduction of inverted ILM flap technique and its many variants widened the surgeons’ *armamentarium* although there is still no consensus as to which technique suits better each and every different clinical presentation^2, 7–9^.

In a previous study we compared “Cover” and “Fill” variants of the inverted ILM flap technique and obtained similar closure rates and visual results except for very large MHs over 700 µm that closed more often when the “Fill” technique was used^3^. Since smaller than 700 µm MHs represent by far the vast majority of cases and seemed to close equally well, we measured retinal sensitivity and fixation stability in an effort to highlight possible advantage of one technique over the other^3^.

The two groups appeared well balanced at baseline (Tables 1 and 2) and anatomic success was achieved in all cases, regardless to surgical technique. At 1-month follow-up, BCVA improved significantly and similarly in both groups from 0.70 at baseline to 0.22 LogMAR; at 3 months the Cover Group showed further improvement to 0.15 LogMAR, while the Fill Group did not (0.22 LogMAR). As a result, visual acuity of the Cover Group at 3 months was significantly better than Fill Group (Fig. 1 and Table 2).

Not surprisingly, BCVA results of both groups overlap most published series whereas the advantage of Cover over Fill Technique at 3 months appears interesting and deserves further scrutiny^1^.

Retinal sensitivity within 4° and 10° behaved accordingly, equally rising from baseline to 1-month follow-up in both Groups (Table 2) while only the Cover Group further improved at 3-months, reaching a significant advantage. Wang et al. also reported a significant increase in retinal sensitivity after MH surgery and found that average retinal sensitivity measured at the very MH rim yielded the highest correlation to final BCVA, similarly to our result within the central 4°^10^.

Fixation stability throughout follow-up improved in differently across the two groups, yielding a significant difference of time and surgery interaction that clearly favors Cover over Fill technique (Table 2) in agreement with BCVA and retinal sensitivity data and offering interesting thoughts. Sun et al. correlated fixation behavior to final visual outcome, suggesting smaller holes have higher peri-lesion sensitivity and are also more prone to recover^11^. Our series does not seem to suggest a prognostic value for fixation stability at baseline but it was not powered to detect it; more patients within the Fill Group showed stable fixation at baseline but more patients moved from unstable to stable fixation in the Cover Group throughout the study follow-up. Unstable fixation may therefore represent a pre-requisite favoring subsequent creation of a stable fixation, a residual plasticity allowing function improvement. Previous studies associating the amount of fixation shifting to BCVA increase also corroborate this hypothesis^12, 13^.

As already mentioned, Rossi et al.^3^ in a previous study found a faster visual recovery and reduction of ellipsoid defect line for the Cover Group but BCVA was eventually similar among Cover and Fill Groups at 3 months.

Remarkably, that surgical series included significantly wider MHs with 550 µm average diameter, 15/26 (57%) over 500 µm and 4/26 (15%) over 700 µm, while this study only included 8/28 (28.5%) MHs greater than 500 µm and an average MH diameter of 397 µm (Supplementary Table S1). Comparing the two studies with significantly different average MH diameter might explain the different outcome and suggest a targeted surgical strategy.

While both Cover and Fill techniques provide a layered scaffold to glial cells migration and proliferation that probably represents the essential plus of all ILM flap variants, the healing mechanism could be conceivably different according to width. Smaller MHs would benefit from ILM “guidance” function of a single layer providing “roof” to a re-created *niche* environment where glial cell slide and growth factors pool. That single layer might be enough to promote bridging of smaller retinal tissue dehiscence without interfering with outer retinal layers healing process and therefore limited or no disruption of their restored architecture. This undoubtedly would give way to a better functional outcome.

MHs larger than a critical and unknown width, possibly over 550–600 µm, on the other hand, might present too voluminous voids to be closed through a simple “roofing” and “sliding” mechanism and may require sheer volume restoration along with the recreation of a closed environment. The Fill technique would provide both, in the shape of an ILM “plug” at the price of losing outer retinal layers continuity thus hindering at least partially the visual recovery. Schubert et al. demonstrated that slopes, steps, and gaps represent insurmountable obstacles to the migration of glial cells, thus the ILM flap would act as a slide and plug in the Fill technique^14^.

Although meta-analysis of small, different and retrospective studies is dangerous and sometimes misleading, it should be noted that present series and previous paper differ significantly in MH size (Supplementary Table S1)^3^. Combining results seems to suggest that smaller MHs show a better visual outcome when Cover technique is used although this affirmation needs to be taken with extreme caution even if both series were operated by the same surgeons with the same techniques.

The main limitation of this study was the relatively small sample size. In addition, in the majority of patients included MHs were smaller than 500 µm.

In summary, there may be a trade-off between the capability of restoring a more physiologic environment capable of guaranteeing a better visual outcome that the Cover technique might offer and the advantage of closing bigger holes proper of the Fill technique. Both techniques have pros and cons since the former seems inadequate for larger MHs, possibly for mere architectural reason while the latter most likely interferes with outer retinal layer restoration thus impacting visual outcome.

Corroborating a similar hypothesis will necessarily require the design of further studies.

## Materials and methods

### Selection of the patients and examination

This study was formally approved by the Central Ethical Committee for Lazio, Italy (protocol no. 14738/December 18th, 2018) and written informed consent was obtained from all participants. All methods were carried out in accordance with relevant guidelines and regulations. All figures and images were made and processed using the GIMP free online software.

The clinical trial “Inverted ILM-flap Techniques Variants for Macular Hole Surgery: Outcomes Comparison” was registered on ClinicalTrial.gov on 22 October 2019 (Identifier: NCT04135638) and was completed on 31 March 2020.

All patients undergoing PPV for idiopathic MH between January and April 2018 at our Institutions have been randomized to receive an ILM flap with either “Fill” or “Cover” technique.

Patients with MH duration greater than 6 months, myopia exceeding 6 diopters, history of trauma, previous ocular surgery except uncomplicated cataract extraction with in-the-bag IOL implantation, any ocular illness including glaucoma, uveitis, optic nerve pathology, were excluded.

All patients underwent comprehensive eye examination at baseline, one and three months after surgery including patent and manifest refraction with EDTRS charts, anterior and posterior segment biomicroscopy, indirect ophthalmoscopy, SD-OCT (Spectralis, Heidelberg Engineering, Germany) including radial scan centred on MH to measure MH horizontal and vertical diameter, ellipsoid zone interruption and foveal thickness. The measurement of the MH diameters, foveal thickness and ellipsoid zone interruption, in the horizontal and vertical SD-OCT B scan passing through the fovea, was carried out manually by two expert graders, masked for either any clinical information or surgical technique, by means of an internal software tool.

In the horizontal and vertical SD-OCT B scan passing through the fovea and used for the analysis, the integrity of the ellipsoid zone was defined as a continuous hyperreflective line at SD-OCT examination, while an interruption of it was considered as photoreceptor anomaly. In case of multiple ellipsoid zone interruptions, overall width was considered as the line gaping outer sections.

Patients with incomplete chart, lost to follow-up or having low quality OCT images were excluded.

Confocal microperimetry used the MAIA (Centervue, Padua, Italy) in a dimly lit room conditions without pupil dilation. A 68-stimuli Goldmann III stimuli grid covered the central 10° with a 4–2 full threshold strategy and fixation target was a 1° diameter red circle. Mean retinal sensitivity of the central 4° and 10° at baseline, 1 and 3 months after surgery was calculated as well as fixation stability and examination time.

The MAIA measures fixation stability by tracking eye movements 25 times/s and plotting the resulting distribution. MAIA microperimetry software calculates automatically fixation characteristics after placing a landmark in the center of the fovea. Fixation stability (P1 and P2) is measured by calculating the percentage of fixation points (%) located within a distance of 1° and 2°, respectively. Automatic classification of stability bases on the following criteria: (1) if > 75% of the fixation points are located within P1, fixation is classified as “stable”; (2) if < 75% of the fixation points are located within P1, but > 75% of the fixation points are located within P2, fixation is defined “relatively unstable”; and (3) if < 75% of the fixation points are located within P2, then fixation is classified as “unstable”.

The study received IRB approval and was conducted in compliance with the Health Insurance Portability and Accountability Act of 1996 and the tenets of the Declaration of Helsinki.

### Surgical technique

All patients underwent a 25G standard 3-port PPV with posterior vitreous detachment induction (if not already present), ILM staining with 0.25 g/l of brilliant blue-G (Brilliant Peel, Geuder, Germany) and creation of a 360° ILM flap around the MH rim.

Phakic patients underwent combined phacoemulsification with IOL implant in-the-bag.

The two groups differed for ILM flap positioning: in the Cover Group the ILM flap was folded as a single layer to bridge tissue dehiscence during air-fluid exchange while in the Fill Group, multiple layers of ILM were deliberately folded within the loss of tissue before air-fluid exchange, as already described^3^.

All eyes received a mixture of 20% sulfur hexafluoride tamponade and were instructed to position face down for 4 h a day during the first 3 days post-operative.

The randomization process has been locally performed. Enrolled patients were randomized at a 1:1 ratio for PPV associated with Cover Group or Fill Group. The randomization sequence was computer generated, on-site computer system combined with allocations kept in a locked, unreadable computer file that investigators could access only after the characteristics of an enrolled participant were entered.

Patients and the research assistants were blinded to group assignment.

### Statistical analysis

This study was designed to show, as main outcome, the differences of sensitivity and fixation stability between patients operated on for idiopathic MH and randomly assigned to Fill or Cover Groups. Assuming a difference between two groups in variation of fixation stability at 3 months after surgery at least 35%, 14 patients in each group were required for an 80% power and 5% significance.

Continuous variables are reported as either mean and standard deviation (SD) or median and interquartile range (IQR) according to their distribution, as assessed by the Shapiro–Wilk test while categorical variables were summarized as frequencies and percentage.

Results are reported separately for the two groups (Cover and Fill) and we tested the difference significance using Mann–Whitney U test and Pearson chi-square test for continuous and categorical variables, respectively.

A non-parametric ranked based model for repeated measurements was also used to evaluate the effect of each factor (surgical technique and time) and their interaction (surgical technique × time) on BCVA, 4° retinal sensitivity, 10° retinal sensitivity and fixation behaviour^15^. Tukey box plot graphs were produced for the graphic visualization of distributions of BCVA, 4° retinal sensitivity, 10° retinal sensitivity values at each time points, after stratification according to the surgical technique used.

Two-ways ANOVA for independent samples was used in the meta-analysis comparing previous and present surgical series^3^.

To assess inter-observer and intra-observer variability for OCT measurements, intra-class correlation coefficient (ICC) was calculated. All tests were two-sided and a level of statistical significance was set at *p* < 0.05. Analyses were performed using the R software environment for statistical computing and graphics (R, version 3.5.1; https://www.r-project.org/).

## Supplementary information

Supplementary Information.

## Data Availability

The data used to support the findings of this study are available from the corresponding author upon request.
