# Supplementary material for: Inverted ILM-flap techniques variants for macular hole surgery: randomized clinical trial to compare retinal sensitivity and fixation stability
Source: Sci Rep. 2020 Sep 28;10:15832. doi: 10.1038/s41598-020-72774-1 (PMC7522249; doi:10.1038/s41598-020-72774-1)
Supplement: Supplementary file 1 — Supplementary Information. [file 41598_2020_72774_MOESM1_ESM.pdf]

Inverted ILM-flap techniques Variants for Macular Hole Surgery: randomized clinical trial to compare Retinal Sensitivity and Fixation Stability.

Andrea Cacciamani, MD,[*] Aldo Gelso, MD,[†] Marta Di Nicola, PhD,[‡**], Fabio Scarinci, MD, PhD,[*] Guido Ripandelli, MD,[*] Ciro Costagliola, PhD,[††] Tommaso Rossi, MD,[‡‡]

Supp. Table S1 – Meta-analysis of previous and current surgical series of MHs operated with Cover and Fill technique

	**Rossi 2016**	**Present Series**	**p**
MH mean diameter (µm)	555.2	397.5	
Standard Deviation (µm)	155.2	121.3	<0.001
